# Ultrasound-Guided Hydrodissection for Dorsal Scapular Nerve Entrapment: A Technical Report on the Scalene and Scapular Approaches

**DOI:** 10.7759/cureus.98571

**Published:** 2025-12-06

**Authors:** King Hei Stanley Lam, Abdallah El-Sayed Allam, Wai Wah Mark Lai, Daniel Chiung-Jui Su, Yonghyun Yoon, Manal Hassanien

**Affiliations:** 1 Faculty of Medicine, The University of Hong Kong, Hong Kong, HKG; 2 Board of Clinical Research, Hong Kong Institute of Musculoskeletal Medicine, Kowloon, HKG; 3 Faculty of Medicine, The Chinese University of Hong Kong, New Territory, HKG; 4 Morphological Madrid Research Center (MoMaRC), UltraDissection Spain EchoTraining School, Madrid, ESP; 5 Department of Physical Medicine, Rheumatology and Rehabilitation, Faculty of Medicine, Tanta University, Tanta, EGY; 6 Pain Management, Revive Musculoskeletal Pain Centre, Kowloon, HKG; 7 Department of Physical Medicine and Rehabilitation, Chi Mei Medical Center, Tainan, TWN; 8 Orthopedic Surgery, Hallym University Kangnam Sacred Heart Hospital, Seoul, KOR; 9 Department of Orthopaedics, Incheon Terminal Orthopedic Surgery Clinic, Incheon, KOR; 10 Department of Rheumatology, Assiut University Hospital, Assiut, EGY

**Keywords:** chronic neck pain, chronic pain management, dorsal scapular nerve entrapment, interscapular pain, middle scalene, nerve entrapment, technical report, ultrasound-guided hydrodissection, ultrasound-guided nerve block, ultrasound scanning

## Abstract

Dorsal scapular nerve (DSN) entrapment is a common yet often underdiagnosed cause of chronic interscapular and neck pain. Ultrasound-guided hydrodissection has emerged as a minimally invasive and effective treatment, utilizing fluid to separate the nerve from constricting fascial structures. This technical report provides a detailed, step-by-step guide for two primary approaches to DSN hydrodissection: the proximal (scalene) approach and the distal (scapular) approach. The scalene approach targets the nerve within the middle scalene muscle, its most common site of entrapment, while the scapular approach targets the nerve as it courses deep to the levator scapulae and rhomboid muscles along the medial scapular border. We describe the requisite patient positioning, sonographic anatomy, key landmarks for nerve identification, and the in-plane injection technique for each method. A standardized injectate of 10 mL containing triamcinolone acetonide (40 mg) and lidocaine in saline is used for both. This report serves as a comprehensive technical reference for pain physicians and interventionalists seeking to enhance the precision, safety, and efficacy of ultrasound-guided interventions for DSN entrapment syndrome, a common yet underdiagnosed cause of chronic interscapular and neck pain.

## Introduction

The dorsal scapular nerve (DSN) is a motor nerve originating primarily from the C5 nerve root, which courses through the middle scalene muscle before descending deep to the levator scapulae and rhomboid muscles along the medial scapular border [1,2]. Entrapment of the DSN is a frequent cause of unilateral neck and interscapular pain, often presenting with a sharp, burning, or stabbing quality [3,4]. This condition is a common yet underdiagnosed cause of upper back pain [5]. While DSN entrapment can occur in individuals of any age or gender, it is frequently associated with repetitive overhead activities, poor posture, and anatomical variations such as a nerve piercing the middle scalene muscle. It may also present secondary to pathologies like cervical radiculopathy, trauma, or hypertrophy of the surrounding musculature [3,4,6]. Despite its clinical significance, this condition is frequently overlooked, leading to misdiagnosis and ineffective treatment [4].

The evolution of ultrasound guidance has revolutionized the management of peripheral nerve entrapments, allowing for real-time visualization and targeted intervention [7,8]. Ultrasound guidance is particularly valuable for visualizing neural structures and performing precise injections, thereby improving the efficacy and safety of peripheral nerve blocks [7,9,10]. Hydrodissection, in particular, involves the injection of fluid to mechanically separate a nerve from adjacent structures, potentially relieving compression, improving microvascular flow, and reducing local inflammation [10, 11]. This minimally invasive technique has gained increasing attention in pain medicine for treating entrapment neuropathies [5]. For the DSN, two primary injection approaches are described: a proximal approach at the level of the middle scalene muscle and a distal approach in the scapular region [7,9,12]. Innovative techniques that combine hydrodissection of the DSN with injections targeting the surrounding musculature have been described to address complex interscapular pain [13].

The proximal entrapment at the middle scalene is considered the most common site of pathology [3,6,14], whereas the distal approach is often perceived as anatomically simpler and potentially safer due to its distance from critical neck structures [7,9]. However, a clear technical guide comparing these two procedures is lacking in the literature. This technical report details the standardized methodologies for these two approaches, which were employed as interventions in a separate randomized controlled trial (RCT) currently under review [15]. The objective is to provide a detailed, step-by-step description of the ultrasound-guided hydrodissection techniques for both the scalene and scapular approaches to the DSN, serving as a practical reference for clinicians.

## Technical report

General considerations

Patient Population

The technical procedures described below were performed on patients diagnosed with DSN entrapment syndrome, as defined in the associated randomized controlled trial [15]. No healthy volunteers were involved.

Injectate

A standardized volume of 10 mL is prepared by mixing triamcinolone acetonide (40 mg) with 2 mL of 2% lidocaine and diluting the mixture to a total volume of 10 mL with normal saline. This volume was selected based on established technical principles for peripheral nerve hydrodissection to ensure adequate circumferential spread around the nerve, facilitating complete separation from constricting tissues [10,11].

Rationale for Injectate Selection

The injectate for this study was standardized to a mixture of triamcinolone acetonide (40 mg) [16], lidocaine, and normal saline. This formulation was chosen to combine the mechanical benefits of hydrodissection with the potent anti-inflammatory effect of a corticosteroid [16], which is particularly relevant for entrapment neuropathies involving an inflammatory component. Given that the pathophysiology of DSN entrapment often involves a significant inflammatory component, the direct pharmacological action of triamcinolone was selected to rapidly address this element and provide sustained relief.

It is important to note that 5% dextrose in water (D5W) has emerged as a compelling alternative injectate for hydrodissection [8-10]. D5W offers an excellent safety profile, devoid of the risks of tissue atrophy or hyperglycemia associated with corticosteroids, and may work through osmotic mechanisms and stimulation of tissue regeneration [8-10]. The choice between corticosteroids and dextrose often depends on the clinician's preference, the suspected primary pathology (inflammation vs. fascial incompetence), and patient-specific factors (e.g., diabetes). The comparative efficacy of these different injectates for DSN hydrodissection represents an important area for future research.

Needle

A 25- to 27-gauge needle of appropriate length (typically 2 inches for the scalene approach and 2-3.5 inches for the scapular approach, depending on patient habitus) was used.

Technique

An in-plane needle approach under continuous ultrasound guidance was employed for both techniques to maximize safety and precision [10,11].

Objective

The procedural endpoint was the real-time ultrasound observation of the injectate creating a fluid halo, achieving circumferential hydrodissection and separation of the DSN from the surrounding tissue [10,11].

Standardization Note

The technical protocols described below were developed and rigorously standardized as the primary interventions for a concurrent randomized controlled trial [15].

Technique 1: the scalene (proximal) approach

Patient Positioning and Setup 

The patient was placed in the supine position. The neck was slightly hyperextended and rotated 15 to 30 degrees to the contralateral side to optimize exposure of the posterior cervical triangle. A high-frequency linear ultrasound transducer (8-18 MHz) was used.

Sonographic Anatomy and Nerve Identification

The transducer was placed in the transverse plane on the neck to identify the brachial plexus lying between the anterior and middle scalene muscles.

The transducer was slid cranially to systematically identify the cervical nerve roots. The key was locating the C5 nerve root, the primary origin of the DSN: 1) C7 level: identified by the presence of only a prominent posterior tubercle, 2) C6 level: emerges between the most prominent anterior and posterior tubercles, and 3) C5 level: emerges between less prominent but equally sized anterior and posterior tubercles.

The DSN was visualized as it arises from C5 and courses within the substance of the middle scalene muscle. It appeared as a monofascicular, hypoechoic fascicle with a hyperechoic epineural border.

**Figure 1:**
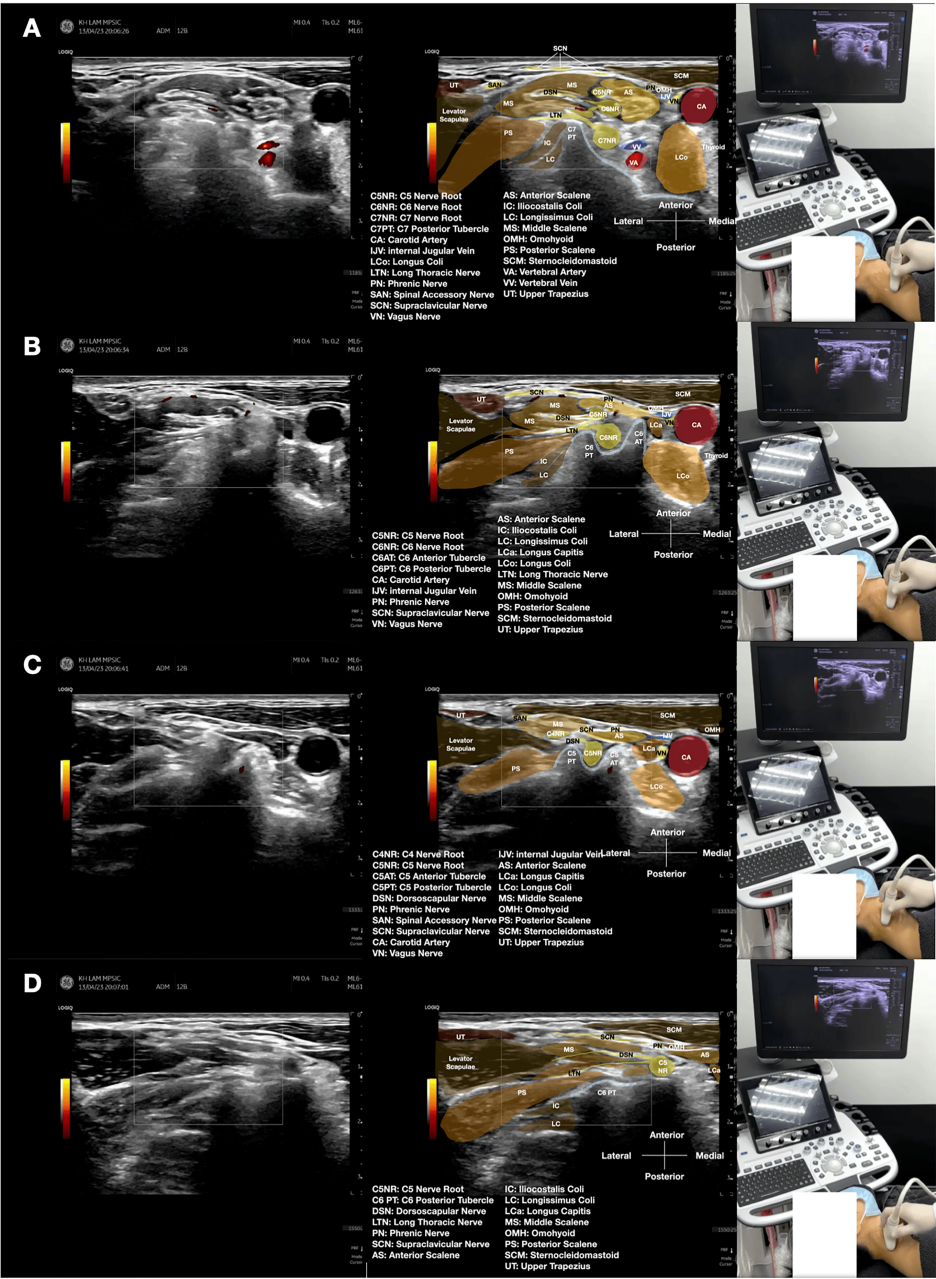
Sonoanatomy of the dorsal scapular nerve in the middle scalene muscle. Ultrasound images demonstrating the anatomical relationships for dorsal scapular nerve (DSN) identification at the scalene level. (A) C7 nerve root level showing characteristic anatomy with a prominent posterior tubercle. (B) C6 nerve root level emerging between prominent anterior and posterior tubercles. (C) C5 nerve root level, the origin of the DSN. (D) The DSN is visualized within the middle scalene muscle as a hypoechoic oval structure (short axis) or fascicular structure (long axis). Key anatomical features include: the middle scalene muscle appearing as a hypoechoic, striated structure; cervical nerve roots as hypoechoic, round structures emerging from intervertebral foramina; and the DSN coursing through the middle scalene muscle (see Video 1).

The nerve was confirmed by tracking it axially within the middle scalene muscle (Figure 1, Video 1).

**Video 1:** Real-time ultrasound identification of the dorsal scapular nerve in the neck. This dynamic ultrasound video demonstrates the systematic approach to identifying the dorsal scapular nerve (DSN) within the middle scalene muscle. The video shows: (1) cranial-to-caudal scanning technique to identify cervical nerve roots C7, C6, and C5; (2) recognition of characteristic bony landmarks (cervical tubercles) at each level; (3) identification of the C5 nerve root as the origin of the DSN; (4) tracking the DSN within the middle scalene muscle; and (5) differentiation of the DSN from surrounding structures in both short-axis and long-axis views. The scanning was performed with a high-frequency linear transducer in the transverse plane.

Hydrodissection Procedure

After standard sterile skin preparation and administration of local anesthesia at the entry point, the needle was inserted in-plane from a lateral-to-medial direction.

Under direct visualization, the needle tip was advanced to the fascial plane immediately adjacent to the DSN within the middle scalene muscle. The target was the potential space between the nerve and the surrounding muscle fibers.

Small test doses of the solution were injected under real-time ultrasound observation. The injectate was observed to begin to separate the nerve from the surrounding muscle fibers. A successful initial injection showed the nerve being lifted away from the tissue.

**Figure 2:**
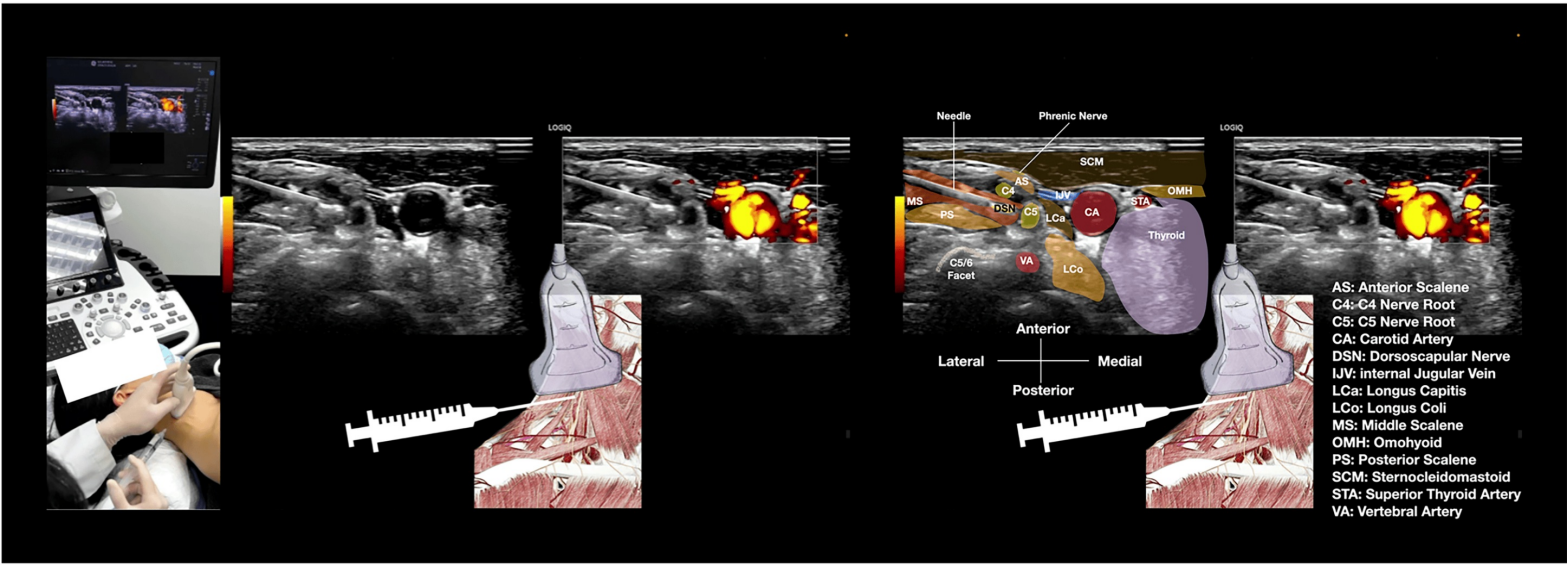
Setup for ultrasound-guided dorsal scapular nerve block via the scalene approach. This schematic illustrates the patient positioning and technical setup for performing a dorsal scapular nerve (DSN) block within the middle scalene muscle using hydrodissection. Key elements include: patient in supine position with neck slightly hyperextended; high-frequency linear ultrasound probe positioned in the transverse plane; 25-gauge, 2-inch needle inserted in-plane from lateral to medial; target DSN visualized as a hypoechoic structure within the middle scalene muscle; and hydrodissection performed with triamcinolone acetonide 40 mg + 2 mL lidocaine (2%) diluted to 10 mL with saline (see Video 2).

Injection was continued slowly to achieve the endpoint of circumferential hydrodissection, where the DSN was visibly surrounded by fluid, indicating successful separation from the constricting scalene musculature. Care was taken to avoid intraneural injection, which is indicated by fusiform swelling of the nerve fascicle (Figure 2, Video 2). 

**Video 2:** Real-time ultrasound-guided hydrodissection of the dorsal scapular nerve via the scalene approach. This dynamic ultrasound video demonstrates the in-plane hydrodissection technique for the dorsal scapular nerve (DSN) within the middle scalene muscle. The video shows: (1) initial identification of the DSN as a hypoechoic oval structure within the middle scalene muscle; (2) needle advancement in-plane from lateral to medial under continuous ultrasound guidance; (3) real-time hydrodissection as the injectate (triamcinolone acetonide 40 mg + 2 mL lidocaine 2% in 10 mL saline) creates circumferential separation of the nerve from surrounding tissues; and (4) confirmation of adequate spread around the nerve. The procedure was performed with the patient in supine position using a high-frequency linear transducer.

Technique 2: the scapular (distal) approach 

Patient Positioning and Setup

The patient was positioned prone. The arms and scapulae were protracted (e.g., by having the patient's hands rest at their sides or placing a pillow under the chest) to open the interscapular space. A high-frequency linear transducer was standard, though a curvilinear transducer was utilized for obese patients.

Sonographic Anatomy and Nerve Identification

The transducer was placed in the transverse plane of the trunk, medial to the superomedial border of the scapula.

The superficial muscles deep to the trapezius were identified: the levator scapulae (attached to the superomedial scapula) and, more caudally, the rhomboid minor (at the scapular spine level) and rhomboid major (below the spine).

The deeper serratus posterior superior (SPS) muscle was identified, which originates from C7 to T3 spinous processes and inserts deep to the levator scapulae and rhomboids, attaching to the second to fifth ribs superficial to the iliocostalis and longissimus muscles.

Power Doppler was activated to locate the dorsal scapular artery (DSA), which runs deep to the levator scapulae/rhomboids but superficial and lateral to the SPS. This artery served as the most reliable landmark for locating the DSN in this region.

**Figure 3:**
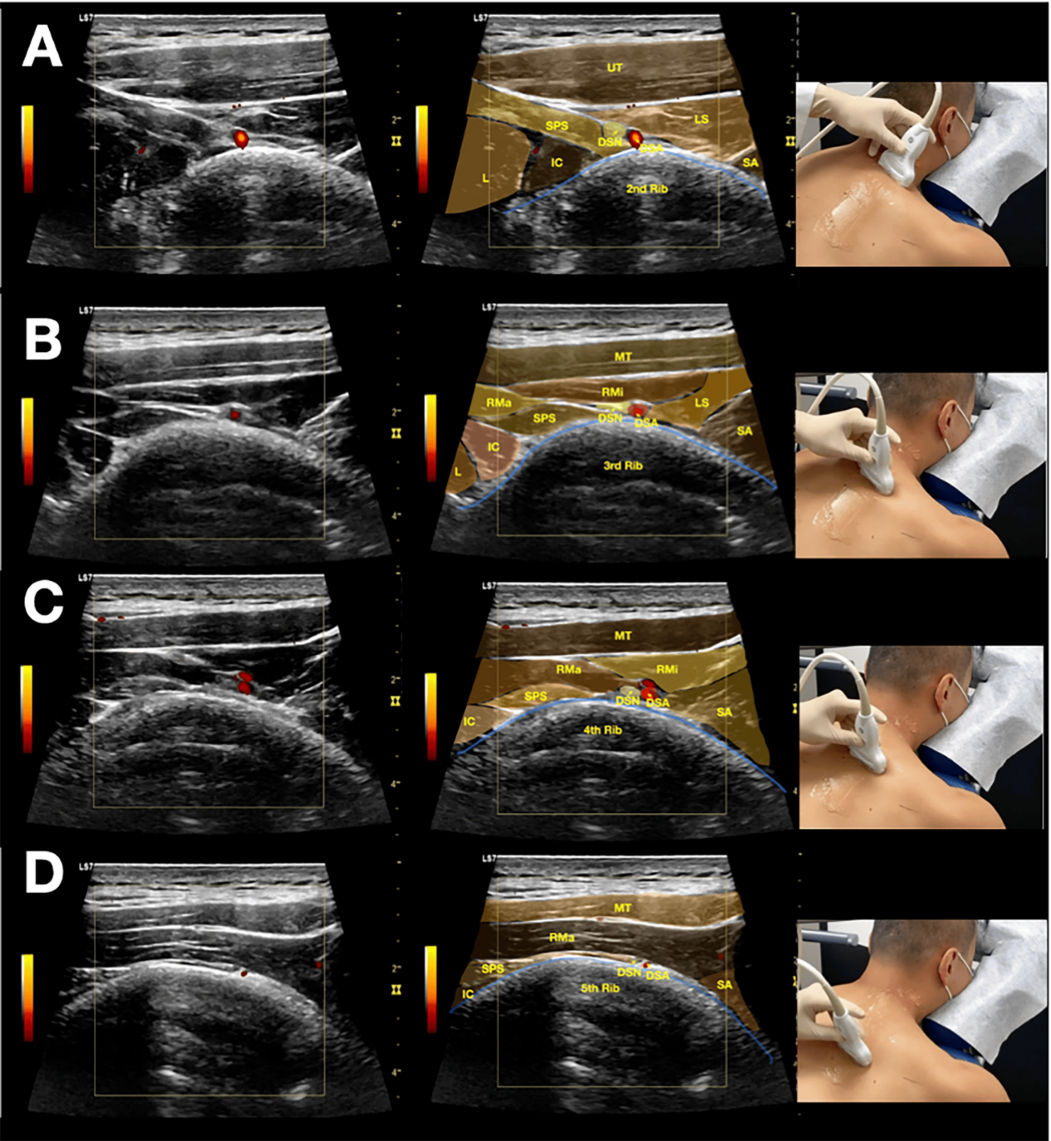
Sonoanatomy of the dorsal scapular nerve in the scapular region. Ultrasound images demonstrating the anatomical relationships for dorsal scapular nerve (DSN) identification in the interscapular region from the second to fifth rib levels. (A) DSN at the level of the second rib. (B) DSN at the level of the third rib. (C) DSN at the level of the fourth rib. (D) DSN at the level of the fifth rib. Key anatomical features include the DSN visualized as a hypoechoic oval structure located deep to the levator scapulae and rhomboid minor muscles, superficial to the serratus posterior superior muscle, and medial to the dorsal scapular artery; superficial muscles appearing as hypoechoic triangular structures; serratus posterior superior as a fan-shaped deep muscle; and ribs as hyperechoic linear structures with acoustic shadowing (see Video 3).

The DSN was typically found as a hypoechoic oval or flattened structure located medial to the DSA, residing in the fascial plane between the deep surface of the levator scapulae/rhomboids and the superficial surface of the SPS. This anatomical relationship is demonstrated in Figure 3 and Video 3.

**Video 3:** Real-time ultrasound identification of the dorsal scapular nerve in the scapular region. This dynamic ultrasound video demonstrates the systematic approach to identifying the dorsal scapular nerve (DSN) along its course in the interscapular region. The video shows (1) medial-to-lateral scanning technique from the second to fifth rib levels; (2) identification of superficial muscles (levator scapulae and rhomboid minor) and deep muscles (serratus posterior superior); (3) use of the dorsal scapular artery as a key vascular landmark; (4) recognition of the DSN as a hypoechoic structure medial to the artery within the intermuscular plane; and (5) dynamic tracking of the nerve across multiple rib levels. The scanning was performed with a high-frequency linear transducer in the transverse plane.

For confirmation, the transducer was rotated to a sagittal plane to visualize the long axis of the DSN as a linear hypoechoic structure running superior to the SPS and the ribs.

Hydrodissection Procedure

After sterile preparation and local anesthesia, the needle was inserted in-plane from a medial-to-lateral direction.

The needle tip was advanced to the plane between the DSN and the fascia between the top of the SPS muscle (deep) and the undersurface of the overlying levator scapulae/rhomboid minor (superficial).

Injection was performed under direct visualization. The injectate was observed to flow to separate the DSN from the surrounding fascial structures. The goal was to see the fluid dissect the plane, pushing the nerve away from the underlying SPS and the overlying muscles, and surrounding the fascicle of the DSN.

**Figure 4:**
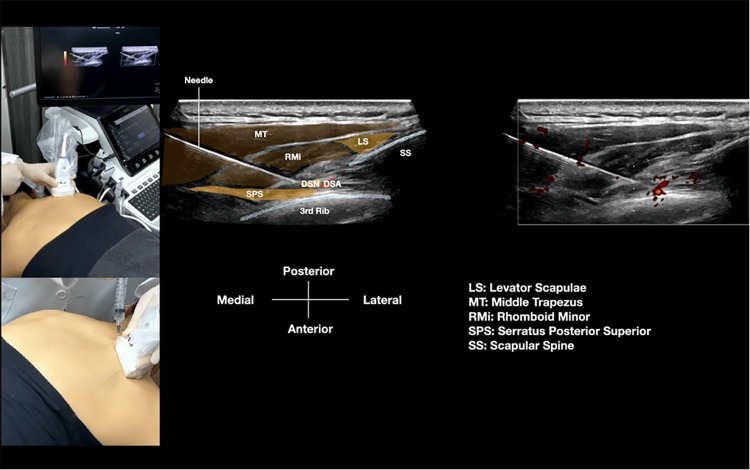
Setup for ultrasound-guided dorsal scapular nerve block via the scapular approach. This schematic illustrates the patient positioning and technical setup for performing a dorsal scapular nerve (DSN) block in the interscapular region using hydrodissection. Key elements include: patient in prone position with arms and scapulae protracted to open interscapular spaces; high-frequency linear ultrasound probe positioned medial to the scapular border; 25-gauge, 2-inch or 22-gauge, 2 3/4-inch needle inserted in-plane from medial to lateral; target DSN visualized as a hypoechoic oval structure deep to the levator scapulae and rhomboid minor muscles, superficial to the serratus posterior superior muscle, and medial to the dorsal scapular artery; and hydrodissection performed with triamcinolone acetonide 40 mg + 2 mL lidocaine (2%) diluted to 10 mL with saline (see Video 4).

The endpoint was the successful hydrodissection of the nerve, creating a fluid space that released the nerve from its adhesions or points of compression (Figure 4, Video 4).

**Video 4:** Real-time ultrasound-guided hydrodissection of the dorsal scapular nerve via the scapular approach. This dynamic ultrasound video demonstrates the in-plane hydrodissection technique for the dorsal scapular nerve (DSN) in the interscapular region. The video shows: (1) identification of key anatomical landmarks including the levator scapulae, rhomboid minor, and serratus posterior superior muscles, and the dorsal scapular artery; (2) visualization of the DSN as a hypoechoic structure medial to the artery; (3) needle advancement in-plane from medial to lateral under continuous ultrasound guidance; (4) real-time hydrodissection as the injectate (triamcinolone acetonide 40 mg + 2 mL lidocaine 2% in 10 mL saline) separates the nerve from surrounding fascial planes; and (5) confirmation of adequate fluid spread around the nerve. The procedure was performed with the patient in prone position using appropriate transducer selection based on the body habitus.

## Discussion

This technical report delineates two standardized methods for performing ultrasound-guided hydrodissection of the dorsal scapular nerve. The fundamental principle of hydrodissection is the mechanical separation of a peripheral nerve from adjacent tissues, which can alleviate compression, break down adhesions, and improve gliding, thereby addressing the core pathophysiology of entrapment neuropathy [10,11]. The growing body of evidence supports its use in various entrapment neuropathies, with its efficacy rooted in both mechanical liberation and potential neuromodulatory effects [11].

The choice between the scalene and scapular approaches involves a careful consideration of anatomy and clinical context. The scalene approach targets the most common site of DSN entrapment [3,6,14]. Proficiency in cervical sonography and precise identification of the C5 nerve root and the DSN within the middle scalene are critical for this technique [14]. This requires a detailed understanding of the DSN's origin and its consistent pathway through the middle scalene muscle [17]. While it requires a steeper learning curve, it directly addresses the primary site of pathology. A significant consideration for the scalene approach is its proximity to the phrenic nerve and the brachial plexus. The scapular approach offers an alternative that may be perceived as safer due to its distance from the pleura and major neurovascular bundles of the neck [7,9]. Its success is heavily reliant on thorough knowledge of sonoanatomy, using the dorsal scapular artery as a consistent vascular landmark to locate the nearby nerve and clear visualization of the fascial plane between the SPS and the levator scapulae/rhomboids. Ultimately, the selection of approach may be individualized based on the suspected site of compression, with the scalene technique targeting a more proximal etiology [18].

The use of a 10 mL injectate volume for both techniques is based on established technical principles for peripheral nerve hydrodissection, aiming to ensure complete circumferential nerve separation [10, 11]. The inclusion of a corticosteroid (triamcinolone) aims to capitalize on the anti-inflammatory effects in addition to the mechanical benefits of hydrodissection. However, it is important to acknowledge that alternative injectates, such as normal saline, have also demonstrated significant efficacy in reducing pain for interscapular conditions, suggesting the mechanical effect of hydrodissection itself is profoundly therapeutic [19,20]. It is important to note that the comparative clinical efficacy of these two specific approaches is the subject of a separate investigation [15].

Safety and potential complications

A thorough understanding of the regional anatomy is paramount to minimizing risks. As with any percutaneous injection, potential complications include local bleeding, hematoma, and infection, though these are rare when using aseptic technique and ultrasound guidance. The overall safety profile of ultrasound-guided peripheral nerve blocks is well-established, with major complications being uncommon when performed by trained clinicians [21]. 

A critical safety consideration for both approaches is the risk of incidental intravascular injection. In the scalene region, the proximity to the vertebral artery and other cervical vessels necessitates careful visualization and frequent aspiration during injection. For the scapular approach, the dorsal scapular artery is intentionally used as a key landmark, making the risk of intra-arterial injection a primary concern. The use of Power Doppler to identify vasculature and the practice of injecting under real-time visualization with small, initial test doses are essential safety measures to prevent this complication.

A further consideration for the scalene approach is the potential for unintended blockade of adjacent neural structures. The 10 mL volume of injectate, containing local anesthetic, may track to involve the proximal C5 nerve root or the upper trunk of the brachial plexus. This can result in temporary motor weakness of the ipsilateral shoulder, manifesting as difficulty with abduction and external rotation. Physicians must warn patients about this potential transient side effect, which typically resolves as the local anesthetic effect dissipates. Pre-procedure counseling should include advising against activities requiring full shoulder strength for several hours post-procedure.

A key challenge with the DSN is its small size, making consistent sonographic identification difficult [22]. This is further complicated by the known anatomical variability in its origin and course, which can include connections to the C6 root or the long thoracic nerve [2]. The systematic scanning protocols described here provide a reliable methodology for nerve localization. As with any interventional procedure, a thorough understanding of the anatomy is paramount to minimize risks. Post-procedure, patients should be monitored for potential complications, such as local bleeding or infection, though these are rare.

## Conclusions

This technical report provides a comprehensive guide for performing ultrasound-guided blocking of the dorsal scapular nerve using hydrodissection techniques via two distinct approaches. The scalene technique targets the proximal, common site of entrapment, while the scapular technique offers a distal alternative. Mastery of both approaches equips the clinician with versatile tools to manage DSN entrapment syndrome effectively. The decision on which approach to employ should be individualized, based on the suspected site of pathology, sonographic visibility, and the operator’s expertise, and a careful consideration of the specific risks and benefits associated with each technique.
